# Association between upper limb movements during drumming and cognition in older adults with cognitive impairment and dementia at a nursing home: a pilot study

**DOI:** 10.3389/fresc.2023.1079781

**Published:** 2023-05-25

**Authors:** Atsuko Miyazaki, Yuichi Ito, Takashi Okuyama, Hayato Mori, Kazuhisa Sato, Masahiko Ichiki, Atsushi Hiyama, Jerome Dinet, Rui Nouchi

**Affiliations:** ^1^ Information Somatics Laboratory, Research Center for Advanced Science and Technology, The University of Tokyo, Tokyo, Japan; ^2^ Computational Engineering Applications Unit, Head Office for Information Systems and Cybersecurity, RIKEN, Saitama, Japan; ^3^ Moff Inc., Tokyo, Japan; ^4^ Department of Public Health, Kobe University Graduate School of Health Sciences, Kobe University, Kobe, Japan; ^5^ Super Reha, LLC., Tokyo, Japan; ^6^ Care 21 Co., Ltd., Osaka, Japan; ^7^ Student and Staff Health Support Center, Tokyo Medical University, Tokyo, Japan; ^8^ Center for the Promotion of Social Data Science Education and Research, Hitotsubashi University, Tokyo, Japan; ^9^ 2LPN (Laboratoire Lorrain de Psychologie et Neurosciences de la Dynamique des Comportements), Université de Lorraine, Nancy, France; ^10^ Department of Cognitive Health Science, Institute of Development, Aging and Cancer (IDAC), Tohoku University, Sendai, Japan; ^11^ Smart Aging Research Center, Tohoku University, Sendai, Japan

**Keywords:** upper limb motor function, upper limb range of motion, drum, acceleration sensors, gyro sensors

## Abstract

**Background:**

Despite the association between motor dysfunction and dementia, quantitative assessment of dementia-related specific motor dysfunction in patients with severe dementia is difficult. Thus, this study aimed to develop a new method to measure upper limb motor function in people with dementia.

**Methods:**

We examined the relationship between dementia severity and dementia-related specific motor dysfunction using the Mini-Mental State Examination (MMSE), a dementia screening test. Participants comprised 16 nursing home residents with a mean age of 86 years and MMSE score of 14.56 (range, 1–23) Points. Participants were seated in a circle and instructed to play a drum that was placed in their lap using mallets (drumsticks) in their dominant hand. Acceleration and gyroscopic sensors were attached to their wrists to collect data on arm movements while drumming. Upper limb motor characteristics were confirmed by recording acceleration and arm movement during drumming and analyzing the correlation with handgrip strength.

**Results:**

Handgrip strength was correlated with arm elevation angle during drumming. The arm elevation angle displayed a significant regression equation with the MMSE score and showed the best regression equation along with handgrip strength (adjusted *R*^2^ = 0.6035, *p *= 0.0009).

**Conclusion:**

We developed a new method using drums to measure upper limb motor function in people with dementia. We also verified that the average arm elevation angle during drumming could predict cognitive dysfunction. This system may be used to monitor people with dementia in a simple and safe way.

## 1. Introduction

Dementia is an umbrella term used to describe a range of neurocognitive disorders. It comprises various symptoms, including a decline in complex attention, executive ability, learning and memory, language, perception (motor and visual perception), praxis, and social cognition (the Diagnostic and Statistical Manual of Mental Disorders, Fifth Edition). The severity of dementia ranges from mild to severe; in severe cases, a significant decline in cognitive function is noted, which can considerably interfere with a person's daily independence (1). Furthermore, people with dementia frequently manifest motor impairments (2). Previous studies reported that motor function decline was highly correlated with cognitive decline (3, 4). Interestingly, the decline in motor function preceded the decline in cognitive function by several years (2, 5, 6). In previous longitudinal studies, a decline in walking speed appeared 12 years before the clinical diagnosis of mild cognitive impairment (MCI) (7) and 7 years before clinical dementia onset (8). Although it is not yet fully described in clinical guidelines (2), accurate characterization of motor impairments associated with dementia could potentially improve diagnostic accuracy (9). Moreover, in one study, the successful management of dementia-related motor symptoms reduced the disability and socioeconomic burden faced by people with dementia and their caregivers (3). Therefore, the accurate characterization of motor decline in the aging population is garnering increasing attention in the research community.

Previous studies reported that upper limb motor function decline was associated with cognitive function decline and dementia (10–13). Handgrip strength is widely used to measure physical and motor functions in the aging population (for example, as an indicator of frailty syndrome), where handgrip strength is related to cognitive function (13). Thus, independent of lower limb function (gait or walking ability), handgrip strength is an important predictor of cognitive impairment (10, 11). Additionally, recent studies have shown that repetitive shoulder movements and elbow flexions are associated with MCI and dementia (12, 14–16). For example, entropy of the elbow angular velocity differed between healthy older adults and older adults with MCI or dementia (12). Furthermore, performances in a dual upper-extremity task (a task of continuously bending the elbow at a steady pace and counting numbers backward) were associated with general cognitive status, as measured by the Montreal Cognitive Assessment (MoCA) (14, 15) and MMSE (16).

Significant correlations have been reported between upper limb motor function and cognitive function in people with dementia. However, upper limb motor function and handgrip strength in people with dementia are practically difficult to measure in the clinical setting. First, handgrip strength and upper limb function are influenced by the patients' health status and healthcare settings because people with dementia are unable to correctly produce motor output owing to apraxia and cognitive decline. The prolonged tenure of residents at nursing homes, which cater to older adults with high care needs, is associated with a rapid increase in physical disability (17), worsening dependence in activities of daily living (ADLs) (18–20), and concomitant poor hand-motor function (21). Second, due to the extent of cognitive decline, people with dementia are sometimes unable to understand and to follow instructions (22). Additionally, people with dementia have several types of apraxia in the early stages of onset (23). Apraxia is a motor dysfunction that occurs in the absence of motor paralysis and includes rudimentary motor, sensory, and language comprehension deficits (24, 25). Some people with dementia are unable to perform motor function tests because of apraxia; in these cases, it is difficult to efficiently measure the motor function. Therefore, it is important to measure motor function that is not affected by apraxia and to conduct motor function tests that can be performed by people with dementia.

Thus, this study developed a new method for measuring upper limb motor function in people with dementia. We focused on drumming movements for several reasons. First, drumming requires active motion, particularly continuous and repetitive movements (shoulder flexion and elbow flexion). When drumming, the biceps raise the upper arms and the triceps pull them down. Therefore, in monitoring drumming movements, we can measure complex upper limb motor function. Second, during actual drumming, the arm can be easily raised with the mallets bouncing off the drum because the striking energy is elastically returned to the player (26). Therefore, people with dementia with weak physical function are still able to perform continuous drumming movements. Third, drumming is a skilled movement that requires a rhythmic response. Rhythmic response function is preserved in patients with severe dementia (27, 28). Thus, people with severe dementia can perform drumming movements efficiently (29).

Our new method is able to measure upper limb motor function in a group setting. There are several reasons why we chose a group setting approach. First, our previous studies demonstrated that even participants with low MMSE scores were able to engage in 30-minute group drumming sessions three times a week for three months without any participant discontinuing due to difficulties arising from the group sessions (29). The second reason is that studies on music therapy for dementia patients are frequently conducted in group sessions (30, 31). The final reason is practicality. It would be useful to measure motor functions during music activities and music therapy in clinical settings. Our goal was to identify motor impairments associated with dementia through the upper limb movements of participants during drum sessions. Utilizing rhythmic auditory stimulation (RAS) (32), which is employed in neurorehabilitation (33), we synchronized motor control and other timing functions during rhythm-based group activities, enabling participants with severe dementia to continue playing the drums. Therefore, we chose the drumming movements in a group setting.

**Figure 1:**
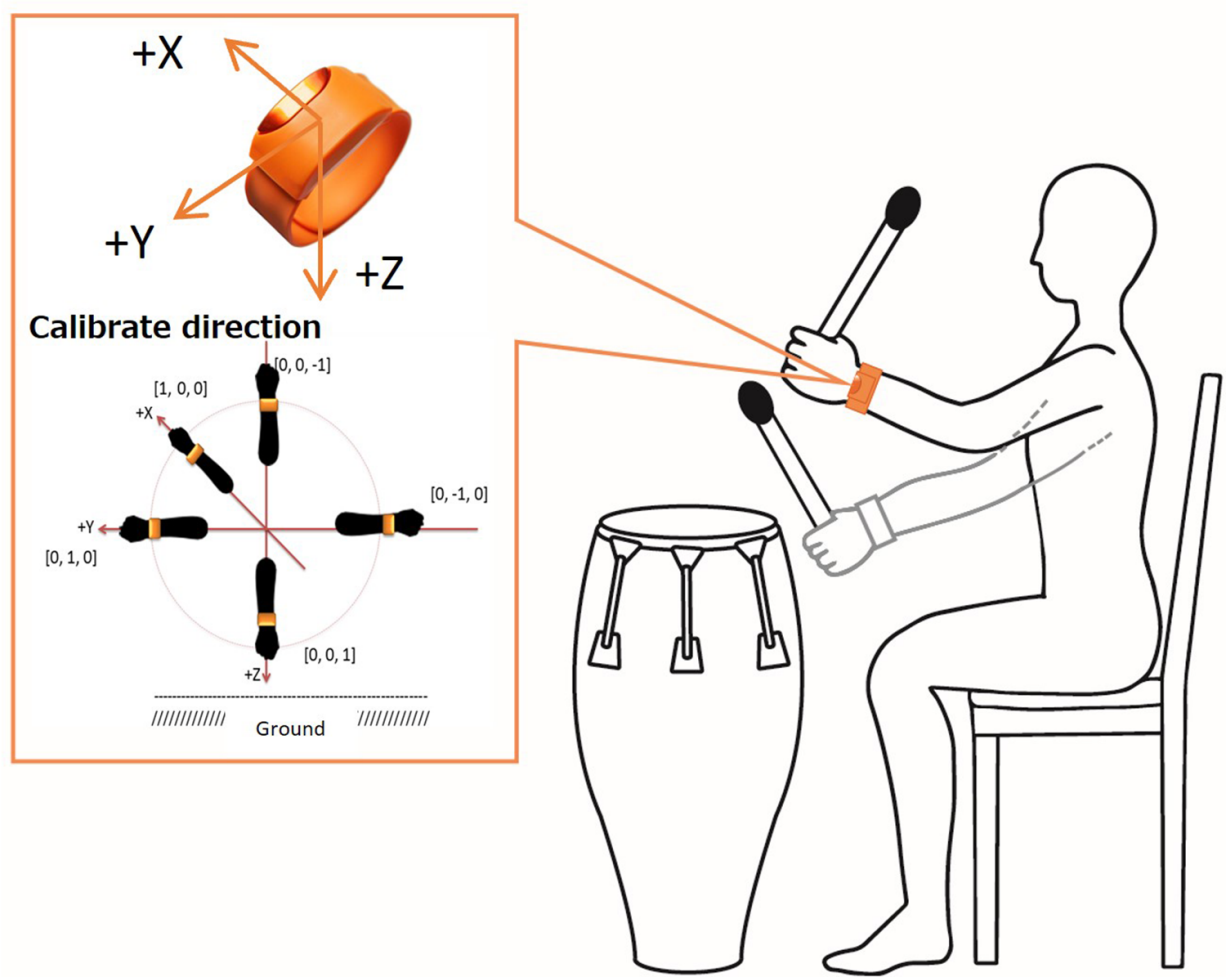
A method for measuring arm movements during a drumming task. The average arm velocity (m/s) and average arm elevation angle (°) are measured from arm movements during drumming using a wristwatch type sensor, with acceleration and gyro sensors attached to the wrist of the dominant arm.

We evaluated the upper limb motor function during drumming movements using wearable accelerometers and gyroscopes, which were attached to the patients' arms. The upper limb motor function was measured by recording the angular velocity and the arm-raising angle while playing the drum. The angular velocity multiplied by the radius of the gyration reflects the average arm velocity in the wristband position. The angle refers to the average arm elevation angle in the wristband position (Figure 1).

This study aimed to develop and to evaluate a new method for measuring upper limb motor function using drumming. We performed two types of analyses: first, we checked the validity of this new method by analyzing the correlation between drumming movements and handgrip performance. We hypothesized that the drumming movements would be directly associated with handgrip performance, as drumming movements require several upper limb functions (26). In the second analysis, we investigated whether the drumming movements would be directly associated with cognitive performance measured using the MMSE. We expected a significant association between drumming movements and cognitive function, as previously reported (12, 16).

## 2. Materials and methods

### 2.1. Participants

We recruited residents from a special nursing home in Tokorozawa City, Saitama Prefecture, Japan. Special nursing homes in Japan provide the highest level of care to older adults outside hospitals for long-term stay toward the end of life.

This study was approved by the Ethics Committee of RIKEN (ref. Wako327-12). We did not estimate a sample size for this study. The number of participants ranged from 10 to 26 in previous studies (12, 14, 15, 34–36). Therefore, 20 participants were recruited in December 2016 for this study. We provided an appropriate study explanation to the participants' guardians, as the participants were unable to directly comprehend our study purpose. The guardians of the participants signed a written informed consent form.

### 2.2. Inclusion and exclusion criteria

The inclusion criterion was the participants' ability to remain seated in a chair for 30 min, as the Drum Communication Program for intervention consisted of 30-min sessions (including the preparatory exercise) (29). The exclusion criteria were apparent severe rheumatism and other joint diseases. In this study, none of the participants met the exclusion criteria.

### 2.3. Demographic characteristics

Details of the participants' demographic characteristics are summarized in Table 1. Despite no information on dementia type among the participants, we assessed the ADLs of older adults with dementia using a scale provided by the Ministry of Health, Labor, and Welfare. This observer-based rating scale has been consistently used in the Japanese long-term care insurance system.

**Table 1:** Participant demographic characteristics and behavioral data.

Criterion	*n* = 16		Woman	Men	*T*-test *p-*value
	Mean (sd)	Range	Mean (sd)	Mean (sd)	
**Demographic characteristics**					
Sex (woman: men)	12:4				
ID-ADL	3.50 (1.03)	2–6	3.50 (1.09)	3.50 (1.00)	1.0000
Age (years)	86.00 (7.81)	72–100	87.00 (8.02)	83.00 (7.26)	.3932
Length of stay (days)	897.50 (673.99)	78–1,730	1,092.50 (668.77)	312.50 (151.58)	.0400
Body height (cm)	150.39 (9.08)	137–174	146.94 (5.71)	160.75 (10.11)	.0037
Body weight (kg)	45.94 (9.55)	27.8–64.1	43.11 (8.26)	54.45 (8.78)	.0343
BMI (kg/m^2^)	20.16 (3.00)	13.90–25.70	19.90 (3.38)	20.95 (1.48)	.5633
SMI (kg/m^2^)	5.10 (1.06)	3.15–7.18	4.80 (0.97)	5.99 (0.88)	.0473
Muscle mass of the dominant upper limb (kg)	1.42 (0.48)	0.72–2.60	1.22 (0.28)	2.04 (0.43)	.0000
**Cognitive function measures**					
MMSE score	14.56 (6.89)	1–23	13.92 (7.46)	21.25 (7.68)	.1129
**Arm movement while drumming**					
Average arm velocity (m/s)	0.60 (0.49)	0.003–1.725	0.67 (0.5)	0.37 (0.43)	.3071
Average arm elevation angle (°)	14.73 (10.32)	−4.29–32.46	14.50 (11.2)	15.42 (8.45)	.8838
**Motor function measures**					
Dominant handgrip strength (kg)	11.44 (6.40)	0.00–24.50	8.82 (3.72)	19.30 (6.66)	.0013
Dominant active shoulder flexion (°)	116.56 (27.85)	70–160	112.92 (28.56)	127.50 (25.98)	.3827
Dominant passive shoulder flexion (°)	137.50 (18.71)	105–170	138.33 (18.75)	135.00 (21.21)	.7693

Values are presented as means (SDs), unless stated otherwise. sd, standard deviation; ID-ADL, activities of daily living of older adults with dementia scale; BMI, body mass index; SMI, skeletal muscle mass index; MMSE, Mini-Mental State Examination.

We measured the mean length of nursing home stay, as the length of nursing home stay is associated with the degree of upper limb disuse and higher levels of disuse may limit the upper limb range of motion (ROM) (21).

### 2.4. Body composition measures

Of 16 residents (3 participants were excluded due to measurement device errors), 13 used wheelchairs at all times for mobility. Therefore, using a standard measuring tape [Model SM-01(2M); Matsuyoshi & Co., Ltd., Tokyo, Japan], we measured the height by measuring the distance between the top of the head and base of the heel. For participants who had difficulty in standing, we measured the height in the supine position on a bed. Additionally, we measured the body weight of these participants using a wheelchair scale (PW-650A; Tanita Corporation, Tokyo, Japan); the weight of the wheelchair was subtracted from the total weight (body and wheelchair). In addition, we measured the body mass index (BMI); kg/m^2^), as excess weight is known to adversely affect executive function, attention, memory, and overall cognition (37) and high BMI (≥25 kg/m^2^) is associated with a risk of moderate-to-severe cognitive impairment (38). Conversely, a high BMI in late life (age ≥70 years) lowers the risk of dementia development (39). Furthermore, we used the skeletal muscle mass index (SMI; kg/m^2^) as a measure of both muscle mass and sarcopenia (40). Lower values indicate greater degrees of low muscle mass and sarcopenia. Moreover, compared with healthy participants, cognitively impaired participants aged >80 years had lower SMI measurements (41). Furthermore, in this study, dominant upper limb muscle mass was measured to assess arm movements during drumming using the dominant hand. We measured these body compositions by bioelectrical impedance analysis (BIA) using InBody S10 (Biospace Co. Ltd., Seoul, South Korea). Because InBody S10 can be used in various body positions, measurements were taken in the supine or seated position, depending on the participants' health condition. All participants' body composition measures were obtained within one month prior to the commencement of the drumming task.

### 2.5. Cognitive function measures

We measured general cognitive function using the Japanese version of the 30-item MMSE (42). The MMSE has 11 subtests. It is widely used in the clinical setting and remains a helpful tool to screen for and assess dementia severity (1). All participants' MMSE scores were obtained within one month prior to the commencement of the drumming task.

### 2.6. Motor function measures

The motor function of all participants was measured in the wheelchairs used daily. These wheelchairs were selected by the facility staff at the time of admission based on the participants' body shape ability. Seat height ranged from 36 cm to 50 cm, which was the lower limb length plus 5 cm. However, due to the very old participants and joint deformities, the seat height was selected according to the physical characteristics of the participants.

The maximum grip strength (kg) of the dominant hand was measured using a Smedley-type digital dynamometer (Takei D TKK5401; Takei Kagaku Kogyo, Tokyo, Japan).

Due to the large variability due to the choice of protocol for measuring grip strength (43), the basic measurement method, shoulder joint in mild abduction, elbow joint extension 0 degrees, and maximum droop position were maintained. However, as the participants were very old, maximum consideration was given to their physical characteristics due to their joint deformities.

In the grip strength meter, the handle length was adjusted so that the participant could grip with the PIP (Proximal Inter-Phalangeal: second index finger). The participants were guided by a physical therapist and encouraged to grip firmly until the maximum value was obtained. Measurements were performed continuously, and participants were asked to perform at least two maximal force trials with their dominant hand to obtain accurate values. Participants used a goniometer (Frigz Medico Japan K.K., Chiba, Japan) to measure ROM of the dominant upper limb by shoulder flexion in order to examine ROM limitation due to disuse. During ROM measurement, participants were confined off the back of a wheelchair and in a 90-degree trunk position. Passive and active movements were measured; one physiotherapist measured upper limb movement, whereas a second physiotherapist assessed joint stabilization and positioning. All participants' motor function measures were obtained within one month prior to the commencement of the drumming task.

### 2.7. Drumming task and arm movement while drumming

All participants were seated in a circle and played tubular drums, such as djembe, tam-tam, tuvano, tantan, and bia drums, with mallets (drumsticks) in their dominant hand. The drum was positioned at a distance on the participant's dominant hand side which allowed for comfortable playing without compromising their posture. The mallets were specifically adjusted to ensure that the drum was level with the mallets in the participant's hands and could be played with a light swing of the mallets. This was achieved by adjusting both the drum height (between 65 cm and 75 cm) and the mallet length (between 30 cm and 40 cm). Additionally, the mallets were positioned so that participants could hold them easily and maintain sustained arm movement. Consequently, participants were able to freely raise their arms for extended periods of time.

They had to pick up the beat and to play the drums per their preference according to the facilitator's instructions (44). The participants were essentially able to play drums at their own pace. Gradually, participants became aware of each other's playing and maintained a synchronized beat on the drums. To ensure that participants did not forget their role in the drum playing, the facilitator added dynamics and created rhythms while continuing to play with their own drum, while giving eye contact and calling out to participants from the center of the circle.

Due to the apraxia associated with dementia, the learning and sustainment of exercise programs for patients are challenging (29). Therefore, information regarding the amount and type of exercise required for this population is still unclear. However, according to the WHO guidelines for exercise for over 65 years old, aerobic exercise should be performed for at least 10 min (45) and at least 30 min per session (46). The Drum Communication Program (29), which was used in this study, lasted 30 min but included breaks and periods when not all participants were drumming. To measure the effects of the drumming, the 20-minute segment of the original program during which all participants played the drums was selected.

The Moff band, a wrist-worn sensor (Moff Inc., Tokyo, Japan), is equipped with gyro and accelerometer sensors, enabling it to detect and measure changes in posture and body movement. In previous studies, the upper limb movement analysis measurements using the Moff band showed a strong correlation with the widely used optical 3D motion capture system, Vicon (https://www.vicon.com/) (47). Moreover, the evaluation of Moff band indicated high inter-rater reliability and its feasibility for use in remote-based training program evaluations (48). It has also been utilized in the pre- and post-assessment of physical abilities in randomized controlled trials (49).

In our study, +we calculated the average arm velocity (m/s) and average angle (degree) of their arm elevation while drumming. The arm movement measurements were the Moff band, weighing 32 g, with dimensions of 43 mm × 25 mm × 15 mm. It was worn on the wrist of the dominant arm in the direction denoted in Figure 1, similar to a wristwatch. The sensor data were transmitted to an iPad via Bluetooth, and we recorded the three-axis acceleration and angular velocity data with a sampling rate of 20 Hz.

### 2.8. Average arm velocity measures

The average arm velocity (m/s) denotes the average velocity of the sensing device Moff at the wearing site during motion.

Average arm velocity (m/s) = (*a *cos (inner product of successive three-dimensional unit vectors)/unit time) × radius of rotation.

$$=(a\cos\;(V^{t}\cdot V^{t-\Delta t})/\Delta t)\cdot r$$

=(Δθ/Δt)⋅r

The radius of gyration was assumed to be the forearm length, calculated as 223 mm based on the average forearm lengths of men and women from the AIST Human Body Dimensions Database for Japanese (https://www.airc.aist.go.jp/dhrt/91-92/data/list.html).

We removed an acceleration threshold ≤0.1 m/s^2^ from all data during the measurement to avoid the inclusion of non-exercise time in the calculation. A threshold value of 0.1 m/s^2^ was set as the value of acceleration for participants who were not exercising and at rest.

### 2.9. Average arm elevation measures

We calculated the average arm elevation angle (°) using the direction of gravity (*z*-axis) and enforcement time average of the maximum value angle within a set time window (1 s).

$$\begin{array}{c} \mathrm{Average}\;\mathrm{arm}\;\mathrm{elevation}\;\mathrm{angle}=a\;\sin(V^{t}(z))\\ =\theta z \end{array}$$

### 2.10. Behavioral analysis

We determined the correlation coefficients (Spearman's rank-order correlations) and permutation test of Spearman's rank-order correlations using the jmuOutlier package in R to determine whether arm movement while drumming was related to the participants' cognitive function, upper limb function, and/or body composition. Subsequently, a permutation multiple regression analysis was performed with the MMSE score as the dependent variable and the average angle of arm height while drumming, average velocity while drumming, sex, age, and grip strength as the independent variables. The use of permutation tests in these analyses is based on several reasons. Permutation tests do not rely on a specific probability distribution for estimation; instead, *p*-values are calculated based on all possible combinations of the actual data. As a result, unknown population parameters and sampling errors do not affect the *p*-values. In this sense, permutation tests can provide exact *p*-values (50). Moreover, permutation tests are a representative resampling method (51) and can correct for type 1 errors (false positives) (52), even in cases with small sample sizes (53). Additionally, Bonferroni methods (54) and Benjamini and Hochberg (False discovery rate; FDR) (55) are representative multiple testing correction methods that control type 1 errors. However, the Bonferroni method may lead to unacceptable levels of type II (false negative) errors (56), and the FDR method may select more false positives (57). Therefore, when using a small sample size, the permutation test is suitable for examining effectiveness (57–60). We performed the permutation multiple regression analysis using the ImPerm package in R to investigate an association between the MMSE score of each participant and correlation measures (61). To check for multicollinearity problems, all measures were put into one multiple regression equation to obtain the variance inflation factor (VIF) values for the explanatory variables. The Akaike information criterion (AIC) was used to check the quality and suitability of the equation model explaining the MMSE. The value of *β* was analyzed by standardizing each indicator. Statistical significance was set at *p *< 0.05 (one-sided) because previous studies showed that higher cognitive function equated to better upper limb motor function (12, 14–16, 34–36). Notably, all analyses were performed in R version 4.1.0 (R Core Development Team, Vienna, Austria, 2021).

## 3. Results

### 3.1. Demographic characteristics

We initially enrolled 19 participants; however, there were three participants for whom we were unable to collect data because of device errors. Thus, 16 participants, comprising 12 women and 4 men with an average age of 86 (range, 72–100) years, were included in our final analyses.

The ADLs of older adults with dementia comprise seven categories (I, IIa, IIb, IIIa, IIIb, IV, and M), with higher scores indicating more severe dementia. The participants in this study had I = 0, IIa = 2, IIb = 7, IIIa = 5, IIIb = 1, IV = 1, and M = 0. The mean length of nursing home stay was 897.50 [standard deviation (sd) = 673.99] days (Table 1).

The mean length of nursing home stay was significantly longer for women than for men (Table 1).

### 3.2. Body composition

The mean BMI values were 19.90 (sd = 3.38) kg/m^2^ and 20.95 (sd = 1.48) kg/m^2^ for women and men, respectively. The mean SMI values were 4.80 (sd = 0.97) kg/m^2^ and 5.99 (sd = 0.88) kg/m^2^ for women and men, respectively. At the Asian Working Group for Sarcopenia 2019, the SMI cutoff values recommended for older adults aged >65 years were 7.0 kg/m^2^ for men and 5.7 kg/m^2^ for women using BIA values (62). The cutoff value for low BMI or low nutritional status was 20 kg/m^2^ (63). Thus, participants were in the underweight zones for BMI values and below the cutoff for the mean SMI value indicating sarcopenia. The mean muscle mass of the dominant upper limb was 1.42 (sd = 0.48) kg (Table 1).

Neither men nor women showed differences in BMI (kg/m^2^), however, men were significantly larger than women in terms of height, weight, SMI, and muscle mass of the dominant upper limb (Table 1).

### 3.3. Cognitive function

Participants scored an average of 14.56 (sd = 6.89) of a maximum of 30 points on the reverse task: four participants had severe dementia (0–10 points), eight had moderate dementia (11–20 points), four had mild dementia (21–26 points), and none had no dementia (27–30 points) [classifications (64) (Table 1).

MMSE scores showed no gender differences (Table 1).

### 3.4. Motor function

The handgrip strength was 11.44 (sd = 6.40) kg. With regard to the grasping task for handgrip strength measurement, the participants had difficulty in understanding the instructions and required more time to perform the task. Ultimately, one participant was unable to perform the measurement.

The degrees of active and passive shoulder flexion were 116.56° (sd = 27.85) and 137.50° (sd = 18.71), respectively. With respect to active movements, participants experienced difficulty understanding the instructions and required a lot more time to perform the task (Table 1).

There was no gender difference in the degrees of active and passive shoulder flexion, however, the handgrip strength was significantly higher in men than in women (Table 1).

### 3.5. Arm movement while drumming

The mean arm velocity while drumming was 0.60 (sd = 0.49 m/s. The mean arm elevation angle while drumming was 14.73° (sd = 10.32). All participants were able to perform the drumming task (Table 1).

No gender difference was found between mean arm velocity and mean arm elevation angle while drumming (Table 1).

### 3.6. Correlation between arm movement while drumming and handgrip strength

To investigate whether the indicator for arm movement during drumming reflects handgrip strength, Spearman's rank correlation analysis was performed for each measure. The average arm elevation angle while drumming tended to correlate with handgrip strength [Spearman's rho (*ρ*) = 0.3528, *p *= 0.0901, permutation test *p*′ = 0.0869]; however, the average arm velocity while drumming showed no significant correlation with handgrip strength (*ρ* = 0.0339, *p *= 0.4504, permutation test *p*′ = 0.4496) (Table 2).

**Table 2:** Correlation coefficients (Spearman's rank-order correlations) and the permutation test of Spearman's rank-order correlations for All Measurements.

	Age	Length of stay	MMSE score	Average arm velocity	Average arm elevation angle	Handgrip Strength	Active shoulder flexion	Passive shoulder flexion	BMI	SMI	Muscle mass of the dominant upper limb
Age	1										
Length of stay (days)	0.1600 *p *= 0.2770	1									
	*p*′* *= 0.2753										
MMSE score	0.2092 *p *= 0.2184	−0.2087 *p *= 0.2190	1								
	*p*′* *= 0.2156	*p*′* *= 0.2165									
Average arm velocity (m/s)	−0.0637 *p *= 0.4074	−0.2399 *p *= 0.1855	−0.0354 *p *= 0.4483	1							
	*p*′* *= 0.4071	*p*′* *= 0.1863	*p*′* *= 0.4511								
Average arm elevation angle elevation (°)	0.4264 *p *= 0.0498*	−0.4341 *p *= 0.0465*	0.5925 *p *= 0.0078*	0.1353 *p *= 0.3085	1						
	*p*′* *= 0.0484*	*p*′* *= 0.0460*	*p*′* *= 0.0080*	*p*′* *= 0.3063							
Dominant handgrip strength (kg)	−0.2942 *p *= 0.1344	−0.4254 *p *= 0.0502†	0.5473 *p *= 0.0141*	0.0339 *p *= 0.4504	0.3528 *p *= 0.0901†	1					
	*p*′* *= 0.1329	*p*′* *= 0.0503†	*p*′* *= 0.0150*	*p*′* *= 0.4496	*p*′* *= 0.0869†						
Dominant active shoulder flexion (°)	−0.2236 *p *= 0.2026	−0.34269 *p *= 0.0969†	0.3891 *p *= 0.0682†	0.5668 *p *= 0.0110*	0.2052 *p *= 0.2230	0.5459 *p *= 0.0144*	1				
	*p*′* *= 0.2000	*p*′* *= 0.0947†	*p*′* *= 0.0677†	*p*′* *= 0.0111*	*p*′* *= 0.2216	*p*′* *= 0.0167*					
Dominant passive shoulder flexion (°)	0.0772 *p *= 0.3881	−0.0790 *p *= 0.3856	0.3903 *p *= 0.0675†	0.2130 *p *= .2142	0.2294 *p *= .1964	0.4425 *p *= 0.0431*	0.6248 *p *= 0.0048*	1			
	*p*′* *= 0.3891	*p*′* *= 0.3837	*p*′* *= .0666†	*p'*= 0.2074	*p*′* *= .1951	*p*′* *= 0.0419*	*p*′* *= 0.0050*				
BMI (kg/m^2^)	0.0178 *p *= 0.4740	0.1104 *p *= 0.3421	0.1268 *p *= 0.3200	0.1147 *p *= 0.3363	0.2118 *p *= 0.2149	0.3114 *p *= 0.1202	0.1904 *p *= 0.2400	0.2577 *p *= 0.1677	1		
	*p*′* *= 0.4728	*p*′* *= 0.3432	*p*′* *= 0.3206	*p*′ = 0.3340	*p*′* *= 0.2131	*p*′* *= 0.1150	*p*′* *= 0.2383	*p*′* *= 0.1629			
SMI (kg/m^2^)	−0.2517 *p *= 0.1736	0.0015 *p *= 0.4979	0.1857 *p* = 0.2456	0.0294 *p *= 0.4586	0.1206 *p *= 0.3282	0.2583 *p *= 0.1671	−0.0472 *p *= 0.4311	−0.3277 *p *= 0.1077	0.3971 *p *= 0.0645†	1	
	*p*′* *= 0.1659	*p*′ = 0.4990	*p*′* *= 0.2432	*p*′* *= 0.4613	*p*′* *= 0.3289	*p*′* *= 0.1676	*p*′* *= 0.4323	*p*′* *= 0.1072	*p *= 0.0634†		
Muscle mass of the dominant upper limb (kg)	−0.2550 *p *= 0.1703	−0.0553 *p *= 0.4195	0.3734 *p *= 0.0771†	−0.1767 *p *= 0.2563	0.1267 *p *= 0.3201	0.6061 *p *= 0.0064*	0.1397 *p *= 0.3030	−0.0522 *p *= 0.4239	0.6274 *p *= 0.0046	0.7629 *p *= 0.0003*	1
	*p*′* *= 0.1702	*p*′* *= 0.4176	*p*′* *= 0.0768†	*p*′ = 0.2557	*p*′* *= 0.3212	*p*′* *= 0.0068*	*p*′* *= 0.3001	*p*′* *= 0.4209	*p*′* *= 0.0061	*p*′* *= 0.0000*	

Values are Spearman's rank-order correlation coefficient as Spearman's *ρ* (rho) with *p*-value. *p*′ value is the *p*-value of the permutation test. MMSE, mini-mental state examination; BMI, body mass index; SMI, skeletal muscle mass index.

* *p*′ < 0.05.

† *p*′ < 0.10.

### 3.7. Correlation between cognitive function, arm movement while drumming, and handgrip strength

The MMSE scores were found to be significantly positively correlated with arm elevation while drumming (*ρ* = 0.5925, *p *= 0.0078, permutation test *p*′ = 0.0080) and handgrip strength (*ρ* = 0.5473, *p *= 0.0141, permutation test *p*′ = 0.0150). However, the MMSE score did not significantly correlate with the average arm velocity while drumming (*ρ* = 0.0339, *p *= 0.4504, permutation test *p*′ = 0.4496) (Table 2).

### 3.8. Prediction of cognitive dysfunction by arm movement while drumming

The presence of multicollinearity among independent variables was checked by the VIF, and all of those values were <5 (Table 3), indicating that there was no problem in the model. Next, the quality and suitability of the equation model explaining the MMSE were examined by the AIC. The AIC values were small for the average arm elevation angle while drumming and for handgrip strength (Table 4). Therefore, the model that included not only handgrip strength (*p *= 0.0076) but also average arm elevation angle (*p *= 0.0018) was the best model (standardized *β* = 0.4298, *t* = 3.6105, *R*^2^ = 0.6035, *p *= 0.0009).

**Table 3:** Collinearity statistics.

Variables	VIF
Age	1.3958
Sex	2.4300
Dominant handgrip strength (kg)	2.5518
Average arm velocity (m/s)	1.1354
Average arm elevation angle (°)	1.5269

VIF, variance inflation factor.

**Table 4:** AICR for MMSE.

Model									AIC
Age	+	Sex	+	Dominant handgrip strength (kg)	+	Average arm velocity (m/s)	+	Average arm elevation angle (°)	59.96
Age	+	Sex	+	Dominant handgrip strength (kg)	+			Average arm elevation angle (°)	57.96
Age	+			Dominant handgrip strength (kg)	+			Average arm elevation angle (°)	56.05
				Dominant handgrip strength (kg)	+			Average arm elevation angle (°)	54.27

AIC, Akaike information criterion; MMSE, mini-mental state examination.

## 4. Discussion

We investigated upper limb motor function during drumming using wearable sensors attached to the arms of older adults with dementia. The purpose of this study was to develop and to evaluate a new method for measuring upper limb motor dysfunction. The new method using drumming calculated the average arm velocity and average arm elevation angle in the wristband position. Our study yielded three main findings. First, the average arm elevation angle showed a correlation with handgrip strength, which indicates that it is a valid unit of measure to determine upper limb motor function. Second, the average arm elevation angle correlated with the MMSE score. Additionally, the model using the average arm elevation angle and handgrip strength was better in depicting cognitive function measured by the MMSE than other indicators.

The primary finding was that the average arm elevation during drumming was associated with the upper limb motor function in people with dementia. The arm motion during drumming requires elbow elevation using the biceps brachii due to the continuous elbow pull-up motion. In actual drumming, the drumming would require minimal muscular strength to raise an individual's own upper limbs (26), as the mallets are bounced off the drum. In contrast, no relationship was found between the average arm velocity and handgrip strength. Drumming can be performed regardless of muscle output. It is possible that rhythmic functions would affect the average arm velocity during drumming. For instance, even if people have sufficient upper limb muscles, if they do not move their arms at a constant tempo, the average arm velocity would be slow. In this study, we did not measure rhythmic functions or experience with music instructions. Future studies should investigate whether the average arm velocity is associated with rhythmic functions.

The second main finding is that the average arm elevation angle during drumming was associated with general cognitive function. Recent studies have shown that repetitive shoulder movements and elbow flexions are associated with the degree of MCI and dementia symptoms (12, 14–16). For example, performances in a dual upper-extremity task (a task of continuously bending the elbow at a steady pace and counting numbers backward) were associated with general cognitive status, as measured by the MoCA (14, 15) and MMSE (16). Additionally, the task required movement of both front arms (extra- and intra-rotational) in participants with an MMSE score of 0–12 points, which differed between healthy older adults and those with MCI or dementia (12). However, this study first showed that the average arm elevation angle during drumming was associated with dementia severity. Further investigation is required to determine the generalizability of this finding in healthy older adults and individuals belonging to other age groups.

The third main finding is that the model using both the average arm elevation angle during drumming and handgrip strength was better in explaining cognitive dysfunction in older adults with dementia than other indicators. Previous studies reported that handgrip strength alone was associated with cognitive impairment (13) and was an important estimator and predictor of cognitive status in older adults (10, 11), given that handgrip strength requires maximal voluntary mobilization of hand muscles and is involved in cognitive processing (65). However, individuals with dementia are unable to understand and to follow instructions due to cognitive decline or apraxia (22). Contrastingly, drumming can be performed by all participants, despite limitations in motor skills caused by cognitive decline, muscle dysfunction, apraxia, and limited ROM due to disuse in severe dementia (29). The inclusion of the drumming movement index developed in this study can more accurately predict and explain cognitive dysfunction. Therefore, the advantage of characterizing motor impairments associated with dementia is that it enabled us to determine the severity of dementia in an indirect, but simple, manner. Currently, clinicians use neuropsychological tests for simple dementia screening. However, the patients often refuse to undergo the test upon the questions revealing it to be a dementia test (66). In previous studies, neuropsychological testing caused subjects distress (67, 68) and posed a threat to the dignity of older patients (69). Several questions cannot be scored for those with visual or hearing impairments, and the scores may be inaccurate for participants without dementia (70). To reduce patient-level barriers to dementia screening, it is important to increase the variation in assessment methods leading to a person-centered approach (71). Thus, methods that measure upper extremity movement will not only contribute to the early detection of cognitive decline, but will also allow for the humane and dignified treatment of patients with severe symptoms. Moreover, measurement of arm movements during drumming can be performed simply by attaching a wristwatch-sized measuring device in the wristband position. Therefore, it is safe and easy to perform and should be actively used in the future. Based on our findings, the use of both handgrip strength and drumming movements to monitor cognitive status in older adults with dementia could be easily applied in a future clinical setting.

In a randomized controlled trial, compared with a control group, the Drum Communication Program was reported to improve the MMSE scores and upper limb physical function in older adults with dementia (29). RAS induced plasticity in damaged brains through rhythmic entertainment, which is referred to as neurological rehabilitation (33). Moreover, clinicians have used RAS to improve motor performance in neurological diseases and brain injuries. This is because synchronization with external beats restores motor coordination (72, 73). We propose the introduction of the program to update and to monitor information by quantitatively assessing dementia-related motor impairment, which will likely improve the results.

Body composition is sex-dependent, with men reported to be on average taller, heavier (74), and greater total skeletal muscle mass than women (75, 76). Thus, men also show higher values on muscle mass measures such as SMI than women (77). Although there is no research on gender differences in muscle mass of the dominant hand in older adults, as SMI is an index for muscle mass of the extremities, it is easy to infer that males have larger SMI (40). There are also reports on gender differences in muscle strength in older adults, with men having stronger grip strength than women (78, 79). In parallel with previous studies, gender differences in body composition and muscle strength were detected among older adults. In addition, the mean lengths of stay in nursing homes were longer for women compared to men. Women reported longer lengths of stay in institutions than men (80), transitioning from home health care to long-term care in institutions (81). Thus, in this study, general differences regarding gender differences were observed.

However, no differences between men and women were found in the drumming movements, which was the primary outcome of this study. Furthermore, the principal analysis, a permutation multiple regression analysis, was gender-corrected. Therefore, the effects of gender differences on the results of this study were not statistically significant. In addition, there was no difference in the drumming movements between men and women, indicating that even women with lower muscle strength and muscle mass could perform the drumming movements. These findings provide evidence that the index developed in this study may be used regardless of gender and body composition differences. In the future, it is necessary to verify the reproducibility of the results of this study with a larger sample.

Our study is not without limitations. First, we had no data on the type of dementia and severity thereof in each participant, nor the responsible lesion. Researchers have hypothesized that motor deficits, rigidity, slowness, and gait disturbances common to Alzheimer's disease (AD) are extrapyramidal signs. Furthermore, extrapyramidal dysfunction can predict the severity of cognitive impairment, the rate of disease progression, and brain lesion localization (9). Combined with the findings of previous studies, ours could be useful for developing rehabilitation interventions, such as motor impairment assessment by dementia type and preventive interventions to reduce the burden of dementia. Second, we did not examine the dual-tasking nature of drumming. Considering that upper limb motor function during dual-tasking enables the screening of the early stages of AD and MCI (14), combining the results of drumming, a dual task that is possible in severe dementia, could improve screening accuracy.

In conclusion, in this study, we developed a new method using drumming to measure the upper limb motor function in people with dementia. We found that the average angle of arm height during drumming was correlated with handgrip strength and the MMSE score in people with dementia. This suggests that the average angle of arm height is a valid measure for upper limb motor function and is easy and safe to use in monitoring the cognitive status of people with dementia. A rapid and objective screening measure for cognitive function is useful in research and clinical settings and can reduce observer bias and subject stress. In particular, it may influence prevention and treatment strategies by physicians and neuropsychologists. Of course, individual sessions may be more appropriate for some participants, depending on their conditions, and music-based intervention programs can be designed flexibly to accommodate participants with a variety of needs and abilities. However, group drumming sessions, by their nature, allow participants to engage in a sustained manner with sufficient concentration, making them an effective intervention for dementia, as well as for measurement during the session.

## Data Availability

The datasets used and analyzed in the current study are available from the corresponding author upon reasonable request.
